# Development of Arthritis as the Initial Involvement in Adult-Onset Cutaneous Polyarteritis Nodosa: Two Cases and Literature Review

**DOI:** 10.1155/2020/8897358

**Published:** 2020-09-16

**Authors:** Ryota Takamatsu, Yasuhiro Shimojima, Dai Kishida, Yasufumi Kondo, Ken-ichi Ueno, Yoshiki Sekijima

**Affiliations:** Department of Medicine, Neurology and Rheumatology, Shinshu University School of Medicine, 3-1-1 Asahi, Matsumoto 390-8621, Japan

## Abstract

Articular symptoms are commonly present in polyarteritis nodosa (PAN). Meanwhile, they may occur as the initial and main involvement of PAN, raising a concern of a delay in a definitive diagnosis of disease unless the histological evidence is obtained. Herein, we report two cases of cutaneous PAN (c-PAN) in which arthritis developed as the initial clinical episode of disease and we argued, through a review of the literature, the clinical feature of patients presenting with arthritis as the initial symptom of PAN. To our knowledge, only six cases have been reported in the English literature, and all six patients were categorized as having c-PAN. Of those patients, four had arthritis with indicating destructive changes. A median of 9 years elapsed prior to the induction of immunosuppressive treatment despite the fact that the other reviewed cases as well as our two patients, who received treatment significantly earlier, showed no evidence of joint destruction. Taken together, this report suggests that the early induction of therapy based on the definitive diagnosis of PAN may be required for preventing joint disruption even though it is not easy to make a diagnosis of PAN unless biopsied tissue can provide the evidence of necrotizing vasculitis.

## 1. Introduction

Polyarteritis nodosa (PAN) is a systemic necrotizing vasculitis targeting small- and medium-sized arteries. Regardless of whether life-threatening severe visceral disorders are seen, physical impairments ascribable to PAN, such as cutaneous, articular, and/or musculoskeletal involvement, are frequently observed in approximately 50% of patients [1, 2] and can be the cause of disability in daily living. In addition, nonspecific appendicular impairments including arthritis have been recognized as a common general symptom in PAN [1–4]. The diagnosis of PAN requires definitive histology, angiography, and/or combination of clinical findings to meet the diagnostic criteria [1, 3, 5]. However, there is a concern that articular involvement may occur as the initial clinical episode before fulfilling the criteria, resulting in a delay in initiating the appropriate treatment. Herein, we present two cases of patients with cutaneous PAN (c-PAN) in whom arthritis developed as the initial clinical episode. In addition, we reviewed the English literature of adult-onset PAN in which arthritis was the initial episode of disease. In this report, we describe the clinical features of PAN in which arthritis initially develops, through an examination of our cases and a review of the literature.

## 2. Case Reports

**Figure 1:**
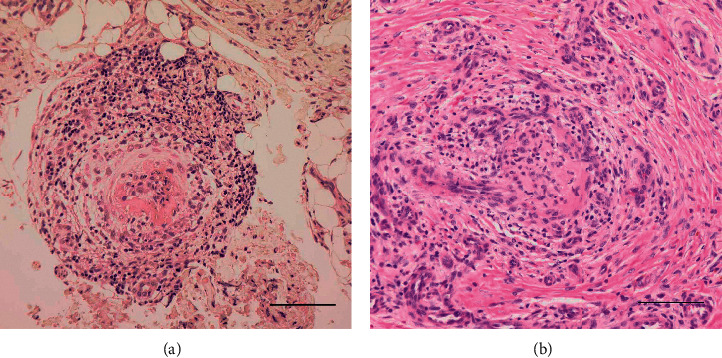
Histopathological findings from synovial tissue (a) and cutaneous tissue (b). The pathological specimens, which were stained with hematoxylin and eosin, display fibrinoid necrosis and luminal occlusion in the arteries as well as inflammatory cell infiltration (scale bar = 100 *μ*m).

A 74-year-old woman, who had suffered from arthralgia with swelling on her right knee joint for nine months, was admitted to our hospital. She additionally had digital ulcers bilaterally which appeared one month before admission. At admission, the patient's temperature was 36.3°C, with swelling and tenderness on the right knee joint as well as ulcers bilaterally on the fingertips. No other physical or neurological manifestations were demonstrated. A systemic survey by contrast-enhanced computed tomography (CT) indicated neither visceral involvements nor visible vascular impairments. In laboratory findings, a urinalysis indicated no abnormality and she had normal values for renal and hepatobiliary functions, as well as for blood count. Additional lab results reported increased levels of serum C-reactive protein (CRP; 7.91 mg/dL; normal, <0.01 mg/dL), C3 (149 mg/dL; normal, 73–138 mg/dL), CH50 (61.2 U/mL; normal, 30–53 U/mL), and erythrocyte sedimentation rate (ESR; 92 mm/h; normal, 2–10 mm/h). The levels of serum C4 was within the normal range (35.2 mg/dL; normal, 17–45 mg/dL). Negative test results included antinuclear antibody (ANA), rheumatoid factor (RF), anticyclic citrullinated peptide antibody (a-CCP), antiphospholipid antibodies (aPL), cryoglobulins, anti-double stranded DNA antibody (a-DNA), anti-Smith antibody (a-Sm), anti-U1 ribonucleoprotein antibody (a-RNP), anti-Ro (SS-A) antibody (a-SSA), anti-La (SS–B) antibody (a-SSB), anti-topoisomerase I antibody, anticentromere antibody, myeloperoxidase (MPO), and proteinase 3- (PR3-) antineutrophil cytoplasmic antibodies (ANCA). The results of the serological tests for hepatitis B (HBV) and C viruses (HCV) were negative. The histopathological finding of synovial tissue biopsied from her right knee joint indicated fibrinoid necrotic vasculitis with inflammatory cell infiltration (Figure 1(a)), resulting in the diagnosis of PAN. She was treated with prednisolone (PSL) at a dose of 60 mg daily after a course of intravenous methylprednisolone administration (1 g daily for 3 days), allowing for achieving clinical improvement. It was ultimately possible for her to maintain remission while tapering the PSL after the administration of methotrexate (MTX).

A 60-year-old woman, with an 18-month history of arthritis bilaterally on the ankle joints, was admitted to our hospital. Her prehospitalization treatment with PSL at 10 mg daily and MTX, which had been initiated 5 months after the onset of the arthritis, was consequently ineffective. She additionally developed erythematous papules bilaterally on her lower extremities 4 months before admission. At admission, the patient's temperature was 36.8°C. There were no other physical or neurological impairments except for tenderness and swelling bilaterally on the ankle joints as well as eruptions on the forearms and lower legs. There were neither visceral nor visible vascular impairments noted on systemic screening by contrast-enhanced CT scan. A urinalysis showed no abnormal findings. The normal values for renal and hepatobiliary functions, as well as blood count, were observed. Elevated levels of serum CRP (4.75 mg/dL), CH50 (73.3 U/mL), and ESR (31 mm/h) were noted. The ANA titer was 1 : 320 (speckled pattern), whereas tests for RF, a-CCP, aPL, cryoglobulins, a-DNA, a-Sm, a-RNP, a-SSA, a-SSB, MPO, and PR3-ANCA were negative. Negative results of the tests for HBV and HCV were indicated. A skin biopsy was performed from the eruption on the lower leg, and the histology demonstrated fibrinoid necrosis and luminal occlusion in the vessels as well as inflammatory cell infiltration in the deep dermis (Figure 1(b)), leading to the diagnosis of PAN. The PSL was increased to 60 mg daily after ceasing the MTX, resulting in prompt improvement of the arthritis and the eruptions. Azathioprine at 50 mg daily was also administered, allowing for tapering the dose of the PSL while maintaining remission.

## 3. Review of the Literature and Our Cases

We eventually reviewed available English articles published between June 1977 and September 2015 through searches of PubMed/MEDLINE and the Web of Science on cases of adult-onset PAN presenting with arthritis as the initial episode of the disease. We used keywords including “arthritis,” “arthropathy,” “PAN,” and “c-PAN.”

The clinical findings in a total of eight patients including the six reviewed cases [6–10] and our two cases are summarized in Table 1. All eight patients were categorized as having c-PAN according to the criteria previously proposed [11, 12]. Of them, seven patients demonstrated the histological findings of necrotizing vasculitis in the cutaneous tissues and one in the synovial tissue. The median duration between the development of arthritis and that of the cutaneous lesions was 4 years (interquartile range (IQR): 1.04–4.75). The median duration of arthritis prior to establishing the definitive diagnosis of c-PAN was 6.5 years (IQR: 1.31–8.5). In the statistical comparison between patients with destructive arthritis (*n* = 4) and those with nondestructive arthritis (*n* = 4) using the Mann–Whitney *U* test, the periods leading up to initiating immunosuppressive treatment based on the definitive diagnosis of c-PAN were significantly longer in those with destructive arthritis than those with nondestructive arthritis (median: 9 vs. 1.13 years; *p*=0.043).

## 4. Discussion

Arthralgia is a common clinical feature in PAN, and approximately 40–60% of patients present with articular symptoms [2, 13, 14]. However, to the best of our knowledge, only six cases were found in the English literature when we focused on arthritis as the initial clinical episode of PAN [6–10] (Table 1). Accordingly, arthritis may uncommonly occur as the initial manifestation in adult PAN, although it has been described that arthritis develops as the first symptomatic episode of childhood PAN in 7.7% of patients [15]. Interestingly, all reviewed cases in the literature were categorized as c-PAN, as were two patients in our report. The category of c-PAN is characterized by limited cutaneous vasculitis based on inflammatory fibrinoid necrosis in small- to medium-sized arteries and often accompanies additional manifestations, including fever, myalgia, arthralgia, and peripheral neuropathy, without any major visceral impairment; therefore, c-PAN has been suggested as a distinct category from the classical systemic form of PAN [11, 16]. In the reviewed cases and the present two patients, arthritis persisted for several years before the development of the cutaneous lesions or the establishment of the definitive diagnosis of the disease. Taken together, it is suggested that arthritis may be one of the initial clinical episodes preceding the appearance of the typical cutaneous lesions in c-PAN. Meanwhile, frequencies of arthritis development were epidemiologically observed in 18–69% of c-PAN cases [11, 12, 16], which is not obviously different in comparison to that seen in the overall population of PAN [1, 2, 14]. Namely, definitive evidence was never found to deny that arthritis can be an initial sign of PAN presenting with major visceral disorders during the overall clinical course, although it has been described that c-PAN rarely develops into the systemic type of PAN [16–18].

Articular destruction was observed in four cases reviewed in the literature (Table 1). They ultimately suffered from arthritis for a median of 9 years before initiation of immunosuppressive treatment based on the diagnosis [7–10], while there were significantly shorter periods before initiating therapy in patients who had no joint destruction in the statistical analysis of Table 1. Accordingly, it was suggested that the long-term persistence of disease activity might lead to the destruction of the joint. Meanwhile, one patient in the reviewed cases presented with nondestructive arthritis although it took 7 years for the initiation of the proper treatment for him [6]. In addition, a previous case of c-PAN also demonstrated the absence of joint destruction even though the patient had rapidly progressive and repeated relapse of arthritis [19]. Persistence of disease activity usually provokes the destruction of targeted joints in autoimmune rheumatic diseases, such as rheumatoid arthritis, psoriatic arthritis, or juvenile idiopathic arthritis, from an early phase of disease. On the other hand, there are some autoimmune diseases, including Behçet's disease, systemic lupus erythematosus, and polymyalgia rheumatica, where the articular involvement is commonly nondestructive. Considering these arguments, we realize that the precise mechanism of developing arthritis remains uncertain in PAN, and the reviewed information is insufficient to clarify the crucial characteristics of patients who display arthritis from an early phase of c-PAN.

The ischemic and inflammatory tissue damage ascribable to occlusion and/or disruption in small- and medium-sized arteries is provoked by immune-mediated responses widely based on excessive induction of proinflammatory cytokines, adhesion molecules, and immunocompetent cells, including macrophages, dendritic cells, and T cells in the development of PAN [1, 20]. Chronic HBV or HCV infection may affect the pathogenesis of PAN [1–3, 21]; meanwhile, neither evidence of HBV nor HCV infection was shown in our 2 patients. Additionally, the underlying pathogenesis is implicated not only in the impairment of target organs but also in nonspecific general symptoms such as fever, myalgia, or arthralgia [1, 3]. Besides, our patient was the only case demonstrating the finding of necrotizing vasculitis in the synovial tissue consistent with PAN despite nonspecific findings in the two reviewed cases (Table 1), suggesting that typical histology results might not be definitively obtained from the synovial tissue. Given the pathological mechanism and clinical results mentioned above, we may consider two hypotheses as follows unless typical histology can be found in synovial tissue. First, arthritis may be the nonspecific synovitis that remotely develops from an involved organ showing typical necrotizing vasculitis. Indeed, it was previously described that arthritis is classified as a comprehensive manifestation of systemic vasculitis which is discriminated from specific-organ involvement showing typical histology [22]. Second, arthritis develops as synovitis based on necrotizing vasculitis, but biopsied synovial specimens may be obtained from outside the target site involving typical pathological findings. It was suggested that deep and/or surrounding portions of involved cutaneous or articular lesions may include the definitive histopathological findings [6]; in fact, our patient's results demonstrated necrotizing vasculitis in the deep dermis, ultimately allowing for the diagnosis of c-PAN. The classification of ANCA-associated vasculitis may be satisfiable in accordance with the criteria of the Chapel Hill Consensus Conference and/or the consensus algorithm proposed by the European Medicines Agency [5, 23, 24]; nevertheless, the definitive diagnosis for both c-PAN and classical PAN necessarily requires histopathological evidence [5, 11, 23]. Accordingly, it is essential that the appropriate biopsy site should be identified for the required typical histology for determining the diagnosis.

In conclusion, arthritis may occur as the initial clinical episode in c-PAN even though only eight cases including our patients have been documented to date. Of them, four patients ultimately presented with articular destruction during a long-term period prior to a definitive diagnosis of c-PAN, whereas other cases, in which treatment was initiated relatively earlier, showed no destruction of joints. It is ultimately suggested that early initiation of a suitable treatment may be required for preventing joint disruption. However, a limited number of cases were reviewed in this report, and thus, an accumulation of cases is necessary for elucidating the definite clinical characteristics of patients presenting with arthritis as the initial involvement of PAN.

## Figures and Tables

**Table 1:** Review of clinical findings in patients with polyarteritis nodosa (PAN) presenting with arthritis as the initial clinical episode.

Author	Age (years)/Sex	Arthritis		Involvement except for arthritis	Duration since arthritis development		Biopsy		Treatment	Outcome
		Location	Property		Skin lesions appearance	c-PAN diagnosis^a^	Site	Histology^b^		
Smukler [6]	66/M	Ankles, knees	Nondestructive	Livedo reticularis and hair loss on the legs	7 years	7 years	Skin	Definite	PSL	NR
							Synovium	Nonspecific	Intraarticular CS	
Steven [7]	39/M	Right wrist, knee, left hip, ankles	Destructive	Erythematous macular rash on the legs	4 years	6 years	Skin	Definite	PSL, SSZ	NR
Flanagan [8]	50/M	Ankles	Destructive	Erythematous papules, ulcers, and patchy violaceous eruption on the legs	4 years	8 years	Skin	Definite	PSL, CPA	PR
							Synovium	Nonspecific		
	51/M	Ankles	Nondestructive	Erythematous nodules and livedo reticularis on the legs; ulcers on the right leg; mononeuritis in the left leg	Simultaneous	5 months	Skin	Definite	PSL, AZA	CR
Horai [9]	60/F	Left ankle, left MTP	Destructive	Ulcers, erythema, and livedo reticularis on the left leg; medium-sized arterial stenosis in the left leg (angiography)	10 years	10 years	Skin	Definite	PSL, TAC	PR
Atzmony [10]	66/F	Left ankle, left midfoot	Destructive	Livedo reticularis, small erythematous nodules, and skin-colored nodules on the legs; ulcers on the left leg; axonal neuropathy on the legs	4 years	12 years	Skin	Definite	PSL, MTX	PR
Our case 1	74/F	Right knee	Nondestructive	Bilateral digital ulcers	8 months	9 months	Synovium	Definite	mPSL, PSL, MTX	CR
Our case 2	60/F	Ankles	Nondestructive	Erythematous papules on the forearms and legs	14 months	18 months	Skin	Definite	PSL, AZA	CR

c-PAN, cutaneous polyarteritis nodosa; PSL, prednisolone; CS, corticosteroid; NR, no remission; SSZ, sulfasalazine; CPA, cyclophosphamide; PR, partial remission; AZA, azathioprine; CR, complete remission; MTP, metatarsophalangeal joint; TAC, tacrolimus; MTX, methotrexate; mPSL, intravenous infusion of methylprednisolone. ^a^All patients were categorized as c-PAN. ^b^The pathological evaluation is determined as “definite” when the biopsied tissue showed the presence of necrotizing vasculitis compatible with polyarthritis nodosa.
